# Delivery Mode Shapes the Composition of the Lower Airways Microbiota in Newborns

**DOI:** 10.3389/fcimb.2021.808390

**Published:** 2021-12-23

**Authors:** Elisa Cardelli, Marco Calvigioni, Alessandra Vecchione, Lisa Macera, Diletta Mazzantini, Francesco Celandroni, Adelaide Panattoni, Mauro Pistello, Fabrizio Maggi, Emilia Ghelardi, Paolo Mannella

**Affiliations:** ^1^ Department of Clinical and Experimental Medicine, University of Pisa, Pisa, Italy; ^2^ Department of Translational Research and New Technologies in Medicine and Surgery, University of Pisa, Pisa, Italy; ^3^ Microbiology Unit, Azienda Ospedaliera Universitaria Pisana, Pisa, Italy; ^4^ Department of Medicine and Surgery, University of Insubria, Varese, Italy

**Keywords:** delivery mode, vaginal delivery, caesarean section, microbiota, lower airways

## Abstract

Radical alterations in the human microbiota composition are well-known to be associated with many pathological conditions. If these aberrations are established at the time of birth, the risk of developing correlated pathologies throughout life is significantly increased. For this reason, all newborns should begin their lives with a proper microbiota in each body district. The present study aimed at demonstrating a correlation between the mode of delivery and the development of a well-balanced microbiota in the lower airways of newborns. 44 pregnant women were enrolled in this study. Microbiological comparative analysis was carried out on tracheobronchial secretions of babies born through vaginal delivery (VD) or caesarean section (CS). All samples showed the presence of bacterial DNA, regardless of the mode of delivery. No viable cultivable bacteria were isolated from the CS samples. On the contrary, VD allowed colonization of the lower airways by alive cultivable bacteria. The identification of bacterial species revealed that *Lactobacillus* spp. and *Bacteroides vulgatus* were the most common microorganisms in the lower airways of vaginally-delivered newborns. Data obtained from quantitative PCRs showed a significantly higher total bacterial load, as well as *Firmicutes* and *Lactobacillus* spp. amount, in VD samples than CS ones, while no statistically significant difference was found in *Torque Teno Virus* (TTV) load between samples. Taken together, our findings confirm the hypothesis that passage through the maternal vaginal canal determines more beneficial colonization of the lower airways in newborns.

## Introduction

Through countless mechanisms of action, the local microbial flora positively interferes with the host’s physiology, by determining protective effects such as the reduction of inflammation (Delgado-Diaz et al., 2019) and infection rate by pathogens (Oscariz and Pisabarro, 2001; Smith and Ravel, 2017), the modulation of the local immune system (Leong and Ding, 2014), the protection from neoplastic transformation (Greten et al., 2004), and the development of organs and systems (Singh et al., 2017). When a severe alteration in the healthy microbiota composition occurs, all or some of these beneficial outcomes fail to cause an imbalance in the regional homeostasis (Li et al., 2019). Subsequently, numerous pathological frameworks may arise. Among them, the onset of infections (Aroutcheva et al., 2001; Witkin et al., 2011; Smith and Ravel, 2017), inflammatory pathologies (Arend, 2002; Matsuzawa et al., 2017; Delgado-Diaz et al., 2019), tumors (Greten et al., 2004), and allergic pathologies (Li et al., 2019) and reduced development of functional organs and immune system (Singh et al., 2017) are the most evident and considerable consequences. If these alterations in the microbiota composition are established at the time of birth or in the perinatal period, subjects may be more susceptible in developing pathological conditions throughout their lives, with a significantly higher occurrence (Sevelsted et al., 2015). For this reason, it is important that all newborns acquire a proper and biodiverse microbiota since birthtime.

According to the so-called “*in utero* hypothesis”, the fetus would already encounter initial microbial colonization at intrauterine level (Collado et al., 2016). Some microorganisms, which may be able to early colonize the fetus’ sterile intestine and presumptively other organs and body districts, can be transmitted from the mother by vertical transmission *via* placenta (Aagaard et al., 2014). Around half of the infant’s intestinal microbiota is likely transmitted at birth, and derives from the vagina (16.3%), intestine (22.1%), oral cavity (7.2%), and skin (5%) of the mother, consisting mainly of *Bifidobacterium* spp. (Ferretti et al., 2018). Thus, the mother’s overall microbiota has been supposed to be important for the infant’s colonization since pregnancy. In addition, it is well known that the mode of delivery influences the composition of the microbial communities residing in different body districts of the newborn and of the adult long-term (Chu et al., 2017). Because of the passage of the child through the cervix and the rich-in-microorganisms vaginal canal, vaginal delivery (VD) confers a different and more stable microbial colonization of the newborn than cesarean section (CS) (Dominguez-Bello et al., 2010). Infants born by VD have microbial consortia of skin, oral cavity, and rectal mucosa more similar to the maternal vaginal microbiota, containing mainly *Lactobacillus* spp., *Prevotella* spp., *Atopobium* spp., and *Sneathia* spp. (Dominguez-Bello et al., 2010). Babies from CS, on the contrary, possess a flora more similar to that residing on the mother’s skin (Dominguez-Bello et al., 2010). Data concerning the gastrointestinal colonization of the newborn show that VD is associated with an increase of *Lactobacillus* spp. and *Bacteroides* spp., while CS with a higher abundance of *Clostridium* spp. (Bäckhed et al., 2015; Stinson et al., 2018). Particularly, a greater abundance of *Lactobacillus gasseri*, a typical member of vaginal flora, has been detected in the intestinal tract of infants from VD (31%) compared to CS (6%) (Nagpal et al., 2016). VD is associated with an increase in *Lactobacillus* spp. also in the upper airways and with the presence of fecal bacteria that are absent in the upper airways of CS infants (Bosch et al., 2016). Moreover, meconium from vaginally-delivered infants contains more DNA sequences associated with *Actinobacteria*, *Gamma-Proteobacteria*, and *Beta-Proteobacteria*, whereas in the CS-derived meconium *Deinococcus*, *Alpha-Proteobacteria*, and *Bacilli* are more abundant (Shi et al., 2018).

Contrary to bacterial populations, the virome, the viral “flora” present in human tissues and not associated with disease, is much less comprehended. However, it’s well-known that eukaryotic viruses commonly reside in healthy individuals, with Torque Teno Virus (TTV), a small single-stranded circular DNA virus, being the most representative and abundant component of human virome (Freer et al., 2018). Increasing evidences demonstrate that TTV can actively interact with the host and probably exert profound effects on human physiology (Maggi et al., 2003). More recently, it has been also suggested that monitoring the intra-host TTV dynamics could be a promising and innovative area of research for characterizing the virus as a new biomarker which would help physicians in the management of patients (Maggi et al., 2018).

As regards the first microbial colonization of newborns, various districts and organs have been analyzed. Nevertheless, no information on the lower airways and the residing microbiota is present in literature. The newborn’s lower airways have never been investigated from the microbiological point of view, although the local microbiota is supposed to play a key role in the correct morphological and physiological development of the mature respiratory system and in the definitive colonization of the newborn. The present study aimed at filling this void of information by analyzing the lower respiratory tract microbiota collected from tracheobronchial aspirates of newborns. A comparison between the microbial colonization following VD and CS was assessed, thus demonstrating a correlation between the mode of delivery and the development of the lower airway microbiota in the newborn.

## Materials and Methods

### Patient Recruitment

Pregnant women (44) were recruited from May 2018 to September 2019 at the Operative Unit of Gynecology and Obstetrics of S. Chiara Hospital in Pisa, according to the following inclusion/exclusion criteria. Only first pregnancies were enlisted and pregnancies of women with ongoing infectious diseases and emergency caesarean sections were excluded. In all examined pregnancies, the rupture of amniochorial membranes occurred less than 12 hours before the delivery. All newborns were born at term (> 37 weeks of pregnancy). Infants with altered airways anatomy or towards who a cardiopulmonary resuscitation at the time of birth was carried out were excluded. In the end, 28 vaginal deliveries (VD) and 16 elective cesarean sections (CS) were considered for this study. The study received Independent Review Board approval (protocol n. 44027). Written informed consent was obtained from the parents of enrolled infants.

### Sample Collection and Processing

Tracheobronchial aspirates from newborn airways were tested as biological samples. Samples were taken within the first 10 minutes of life carefully avoiding any kind of contamination. A sterile disposable suction device connected to a wall aspirator near the neonatal island in the delivery/operating room was used for the collection. Suctioning occurred first at the level of the oral cavity and later through the nose. This procedure is routinely performed by neonatologists to clear airways from amniotic fluid for improving the infant’s respiratory exchanges and to verify airways anatomy (Foster et al., 2017). After being collected, samples were immediately diluted in 10 mL of sterile PBS, vortexed, and then centrifuged at 1,500 rpm for 10 min at 4°C to separate the eukaryotic component from the microbial one. Pellets and supernatants were separately stored at -20°C.

### Bacterial Isolation and Viability Evaluation

Tubes containing the microbial component were thawed and centrifuged at 4,500 rpm for 15 min at 4˚C. Supernatants were discarded and pellets resuspended in 1 mL of cold sterile PBS. Suspensions were vortexed vigorously to get homogenized. 500 µL of the microbial suspensions were 1:2 serially diluted in sterile PBS and aliquots (50 µL) from each dilution were streaked on plates of MacConkey agar (MEUS, Italy), Mannitol Salt agar (MEUS), Chocolate agar (MEUS), Schaedler agar (MEUS), and TSH agar (Trypticase Soy agar + 5% Horse Blood, MEUS). Plates were incubated at 37°C for 24-48h in aerobic as well as anaerobic atmospheres, the latter guaranteed by using AnaeroGen Compact (Thermo Fisher Scientific, USA) anaerobiosis generators. After incubation, the number of colony-forming units (CFUs)/plate was determined. Two technical replicates were carried out for each sample. Arithmetic means of counted CFUs/plate were calculated for each sample and related dilutions and replicates. Starting from these data, two global arithmetic means were determined at last for VD and CS samples, separately. To ensure sterility of reagents and plates and thus an effective contamination control, negative controls obtained by streaking 50 µL of PBS on the different culture media were included in each experiment.

### Molecular Identification by MALDI-TOF MS

Well-isolated and morphologically distinguishable colonies from different agar plates were directly spotted on the MALDI plate, overlaid with 1 µL of 70% ethanol, 1 µL of formic acid, and 1 µL of acetonitrile to obtain the highest proteins’ extraction efficacy. Finally, 1 µL of a α-cyano-4-hydroxycinnamic acid matrix was added onto each spot and air-dried. Once the plate was inserted into the MALDI-TOF Microflex LT Mass Spectrometer (Bruker, USA), mass spectra were automatically acquired at a laser frequency of 60 Hz with an acquisition range from 1.960 to 20.000 Da. Spectra were imported into the integrated MALDI Biotyper software (version 3.1, Bruker) and compared with reference spectra found in its database, thus returning identification scores as results. A score ≥ 2.00 indicated identification at the species level, a score ranging from 1.99 to 1.70 indicated identification at the genus level, whereas any score < 1.70 meant no significant similarity of the obtained spectrum with any database entry. Each colony was tested six times.

### DNA Extraction

QIAamp DNA Mini Kit (QIAGEN, Germany) was used to extract genomic DNA from pellets and supernatants, separately. The extraction procedure was performed following the manufacturer’s protocol. DNA concentration was calculated by measuring the optical density at 260 nm (OD_260_) and DNA purity was estimated by determining OD_260_/OD_280_ and OD_260_/OD_230_ ratio with the spectrophotometer BioPhotometer D30 (Eppendorf, Germany). Quantified samples were then properly diluted in sterile water to reach a standard concentration of 1.5 ng of DNA/µl.

### Real-Time Quantitative PCR

16S rRNA gene-based quantitative PCR (qPCR) analysis of normalized DNAs was used to quantify the amount of total bacteria and microorganisms belonging to *phylum Firmicutes* and *genus Lactobacillus* in the samples. Likewise, the number of DNA copies related to TTV load was also determined. Specific primer pairs used in this study are listed in **Table 1**. All reactions were carried out in triplicate in a 96-wells plate with a final reaction volume of 20 µL, containing 2 µL of 1.5 ng/µl DNA template, 10 µL of Luna Universal qPCR Master Mix (New England BioLabs, USA), 0.5 µL of each primer (0.25 µM), and 7 µL of sterile water. Negative controls obtained by replacing the volume of DNA template with an equal volume of sterile water were included. The genomic DNA from *Lactobacillus rhamnosus* ATCC 7469 was used as positive control in qPCRs to assess the amplification success and specificity. qPCRs were performed by using the CFX Connect Real-Time System (Bio-Rad, USA) and CFX Maestro Software (version 2.0, Bio-Rad). For each pPCR, three biological replicates were carried out on different days. The amplification conditions were as follows: an initial denaturation step at 95°C for 1 min, followed by 45 cycles of denaturation at 95°C for 15 s, annealing at primers' optimal temperature for 30 s (**Table 1**), and extension at 72°C for 10 s (Biagini et al., 2020). To check the amplification specificity, a melting curve analysis was carried out by increasing the annealing temperature from 65°C to 95°C after qPCRs. Serial ten-fold dilutions of external standards with known concentrations ranging from 10^2^ to 10^10^ DNA copies/µl were used to generate calibration curves for the absolute quantification in the samples. As far the standards’ preparation, genomic DNA from *Lactobacillus rhamnosus* ATCC 7469 was extracted and used as template for three separate PCR reactions, each with a primer pair targeting 16S rRNA gene-conserved regions of all bacteria, *Firmicutes*, and *Lactobacillus*, respectively (**Table 1**), following the same protocol of qPCRs. Reaction mix for each PCR amplification contained 1 µL of genomic DNA of *L. rhamnosus*, 5 µL of 5X Wonder Taq Reaction buffer (Euroclone, Italy), 0.5 µL of each primer (0.4 µM) (**Table 1**), 0.5 µL of Wonder Taq (Euroclone), and 17.5 µL of sterile water, for a final volume of 25 µL. The resulting amplicons were subsequently run in a 1% electrophoresis agarose gel and DNA bands corresponding to the amplicons’ molecular weights were excised and purified from gel by using NucleoSpin Gel and PCR Clean-up (Macherey-Nagel, Germany), following the manufacturer’s protocol. These PCR products were subsequently quantified by BioPhotometer D30 (Eppendorf) and separately used as specific external standards in qPCR amplifications.

**Table 1:** Primers used for Real-Time qPCR amplifications.

Investigated *taxon*	Primer name and sequence (5’-3’)	Amplicon length (bp)	Annealing T (°C)	Reference
All bacteria	Eub338F - ACTCCTACGGGAGGCAGCAG Eub518R - ATTACCGCGGCTGCTGG	200	60	(Fierer et al., 2005)
*Firmicutes*	Firm934F - ATGTGGTTTAATTCGAAGCA Firm1060R - AGCTGACGACAACCATGCAC	126	60	(Queipo-Ortuno al., 2012)
*Lactobacillus*	LacF - GAGGCAGCAGTAGGGAATCTTC LacR - GGCCAGTTACTACCTCTATCCTTCTTC	126	63	(Queipo-Ortuno al., 2012)
*Torque Teno Virus*	AMT-S - GTGCCGIAGGTGAGTTTA AMT-AS - AGCCCGGCCAGTCC	63	50	(Fornai et al., 2001)

### Statistical Analysis

Data were expressed as the mean ± standard deviation. All statistically significant differences between VD samples and CS samples were detected by applying Student t-tests for unpaired data. Statistical significance was set at a P-value of 0.05.

## Results

### Microbial Viability Evaluation and Molecular Identification by MALDI-TOF MS

Both VD and CS suspensions were streaked on different solid media. After incubation, the number of CFUs grown on each plate was determined. Thus, a total of 1.05 × 10^5^ ± 6.47 × 10^3^ CFUs/mL was recorded for VD samples. On the contrary, no CFUs were found from plates on which CS samples were streaked, thus suggesting that no viable cultivable bacteria were present in these samples after collection and processing. Isolated colonies from VD plates were subsequently submitted to MALDI-TOF MS for microbial identification. The microbial species identified by mass spectrometry are listed below: *Lactobacillus crispatus* (20 out of 28 VD), *Bacteroides vulgatus* (16), *Staphylococcus epidermidis* (12), *Lactobacillus jensenii* (10), *Escherichia coli* (9), *Bifidobacterium longum* (8), *Bifidobacterium pseudocatenulatum* (8), *Streptococcus mitis* (5), *Streptococcus oralis* (5), *Propionibacterium acnes* (5), *Staphylococcus aureus* (5), *Kocuria kristinae* (5), *Lactococcus lactis* (4), *Corynebacterium coylae* (3), *Parabacteroides distasonis* (3), *Enterobacter cloacae* (3), *Enterobacter ludwigii* (2), *Citrobacter amalonaticus* (1), *Bacteroides coprophylus* (1), and *Acidominococcus intestini* (1). All microorganisms were identified with a score > 2.00.

### Absolute Quantification of All Bacteria, *Phylum Firmicutes* and *Genus Lactobacillus*

**Figure 1:**
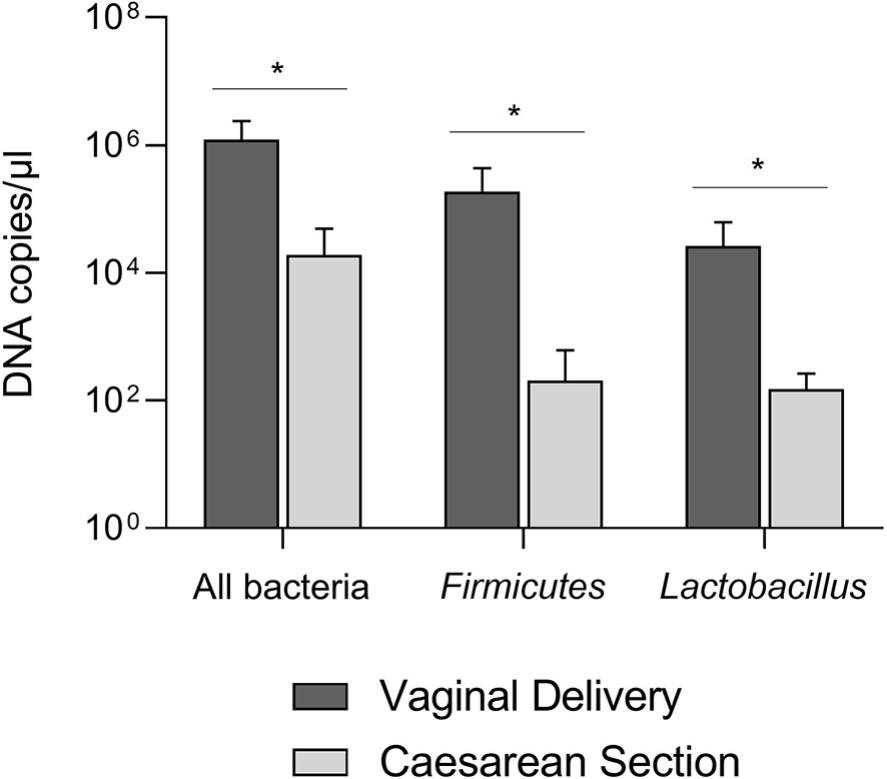
Absolute abundances of the total bacterial load, *Firmicutes*, and *Lactobacillus* in tracheobronchial aspirates from newborns born by vaginal delivery (dark grey) or by caesarian section (light grey). *P < 0.05.

Extracted bacterial DNAs were subjected to Real-Time qPCR to quantify the amount of total bacteria and bacteria belonging to *phylum Firmicutes* and *genus Lactobacillus*. All samples showed the successful amplification of the 16S rRNA gene, even CS samples in which no bacteria were detected in the viability assays, thus confirming the presence of bacteria in both types of aspirates, regardless of the different mode of delivery. As shown in **Figure 1**, the amount of total bacteria found in VD samples was higher than that of CS samples (* P = 0.0124), as well as for absolute abundances of *Firmicutes* (* P = 0.0467) and *Lactobacillus* (* P = 0.0252). Taken together, these overall data demonstrated the presence of endogenous bacterial DNAs in both VD and CS samples and higher abundances of selected *taxa* in VD samples compared to CS samples.

### Absolute Quantification of TTV

**Figure 2:**
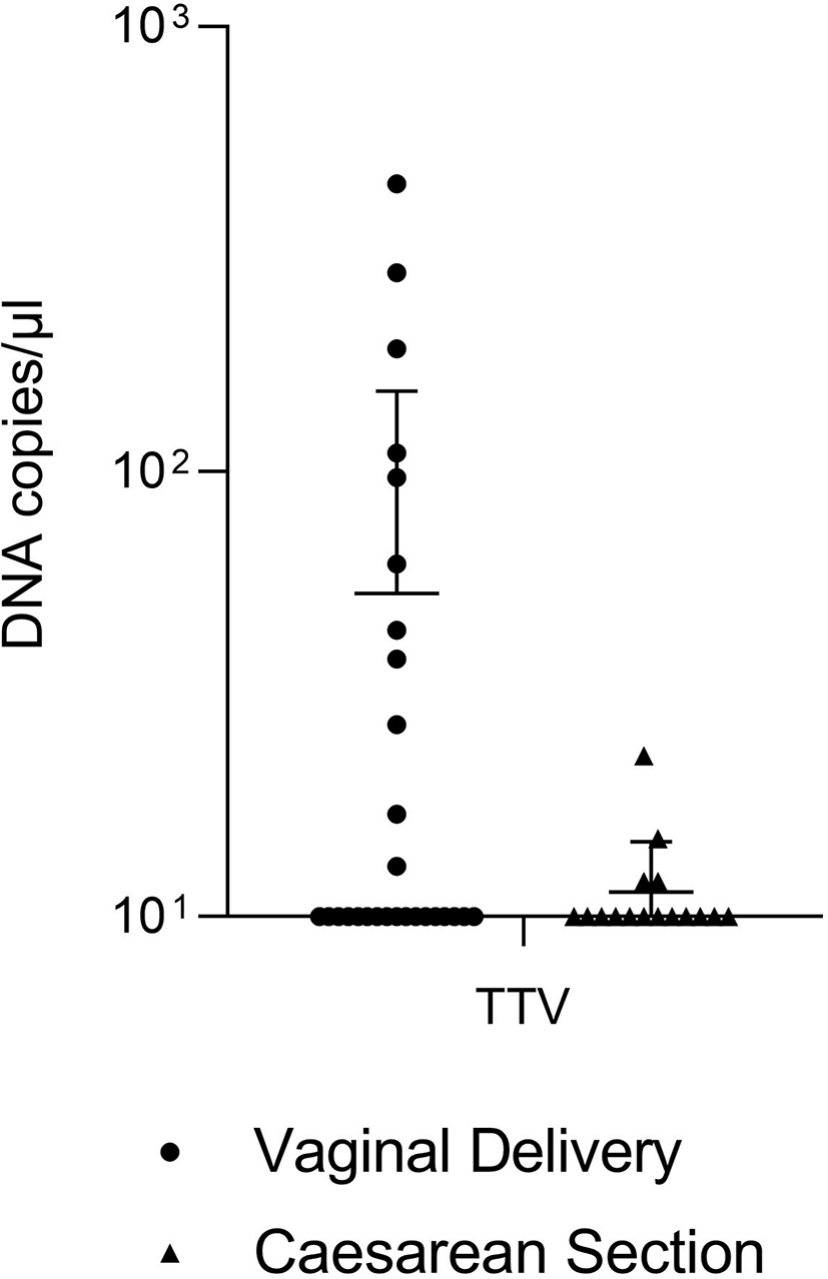
Absolute quantification of TTV in tracheobronchial aspirates from newborns born by vaginal delivery (dots) or by caesarian section (triangles).

Real-Time qPCR was also carried out on DNAs extracted from eucaryotic cells to evaluate the number of DNA copies from TTV (**Figure 2**). Surprisingly, most of the samples contained less than 10^1^ TTV DNA copies/µL (17 out of 28 VD, 12 out of 16 CS). The analysis of remaining samples did not allow to ensure a statistically significant difference in the absolute abundance of TTV between VD and CS (P = 0.0989), although very high titers of TTV (*i.e*. 443 DNA copies/µL, 280 DNA copies/µL) were reached in some VD samples.

## Discussion

This study aimed at inferring a correlation between the mode of delivery and the microbial composition of the infant’s lower airways microbiota. Similar correlation studies have already been performed for the gut microbiota, as well as for microbial communities residing in the upper respiratory tract, in the oral cavity, and on the skin (Dominguez-Bello et al., 2010). However, the microbiota inhabiting the lower respiratory tract has never been studied in association with delivery to the best of our knowledge. As it happens for other body districts, we believe that proper microbial colonization of the infant’s lower airways can be essential for the developing of a well-working respiratory system and for predicting a possible adult’s health/disease condition. In addition to the unusual microbial *consortium* tested, the innovation of this research also lies in the type of sample used for the analysis of the infant’s colonization. Exploiting for this purpose a biological sample (*i.e.* newborn’s tracheobronchial aspirate) that is commonly thrown away by health professionals after its removal from the deep airways may be a novel painless approach to obtain information about the lower airways microbiota in newborns.

As shown by results obtained by qPCR, all VD and CS samples were found positive for the presence of bacterial DNA, thus demonstrating that an initial microbial colonization of lower airways occurs regardless of the mode of delivery. However, finding microbial DNA in a sample does not provide any information about the viability of inhabiting microorganisms. Therefore, samples were also streaked on solid culture media to assess the microbial viability and cultivability, which is an important feature of viable cells. It was surprising to observe that no colonies were grown on agar plates from CS samples, whereas VD samples showed a much different situation. This result suggests that bacterial DNAs found in CS aspirates are derived from non-viable or non-cultivable bacteria. On the contrary, vaginal delivery seemed to allow colonization by viable microbes, which are also presumptively able to carry out their biological functions, thus providing the beneficial effects associated with a healthy flora.

MALDI-TOF MS allowed us to identify the microbial species grown from VD, showing a prevalence of bacteria from *genera Lactobacillus* (*i.e. L. crispatus* and *L. jensenii*), *Bacteroides* (*i.e. B. vulgatus*), *Streptococcus* (*i.e. S. mitis* and *S. oralis*), and *Bifidobacterium* (*i.e. B. longum* and *B. pseudocatenulatum*). In particular, members of *genus Lactobacillus* were the most represented in VD samples collected in this study. Lactobacilli represent a high percentage of the maternal vaginal flora, especially during pregnancy, with *L. crispatus, L. vaginalis, L. jensenii*, and *L. gasseri* as the predominant species (Fox and Eichelberger, 2015; Gabriel et al., 2018). This enrichment occurs because of the increased estrogen levels associated with pregnancy, which determine a higher vaginal glycogen deposition that may promote proliferation of *Lactobacillus* (Gabriel et al., 2018). The increase in *Lactobacillus* spp. is also associated with a contextual reduction of anaerobic species in the vagina (Fox and Eichelberger, 2015). Therefore, the passage of the fetus through the vaginal canal, which occurs during the expulsion phase of childbirth, likely results in the main colonization of the infant’s lower airways by *Lactobacillus*. Moreover, it has been already demonstrated that the vaginal microbiota can be classified into five community state types (CSTs) dependently from the species which is prevalent in the microbial population (Mancabelli et al., 2021). In these vaginotypes, *L. crispatus*, *L. gasseri*, *L. iners*, and *L. jensenii* are the main representatives of CST I, II, III, and V, respectively. Our results, highlighting *L. crispatus* and *L*. *jensenii* as the only representatives of *genus Lactobacillus* inhabiting the VD samples, may be explained considering that the mothers enrolled for the present study casually harbored CST I and CST V in their vaginas, thus drastically reducing the possibilities to find *L. iners* and *L. gasseri* in VD samples.

Subsequently, we assessed the amount of all bacteria and specific bacterial *taxa* in both VD and CS samples. The quantitative analysis of genomic DNAs through Real-Time PCR demonstrated that the total microbial load in tracheobronchial aspirates was significantly higher after VD than CS, thus suggesting that the bacterial flora residing in the deep airways of babies born from VD is quantitatively more abundant. As far as *Firmicutes* and *Lactobacillus* concern, both *taxa* resulted more abundant in VD samples than CS samples. Our choice to evaluate in Real-Time PCR the absolute abundance of *Lactobacillus* just arose from the observation of results obtained in the previous viability experiments. These results were in line with what was previously discussed, confirming a greater infantile colonization by lactobacilli in vaginally-delivered babies than those delivered through caesarean section also in the lower airways.

The effects determined by *Lactobacillus* on the human host have been widely discussed in many *in vitro* and *in vivo* studies. They can produce lactic acid, whose secretion induces a local lowering of pH (Aagaard et al., 2012) and the release of IL-23 by peripheral blood mononuclear cells. IL-23 promotes the maturation of T-cells (Smith et al., 2013) and the release of IL-17, which induces recruitment and activation of neutrophils near the mucosa (Boskey et al., 2001), thus stimulating the local immune system to protect against pathogens (Witkin et al., 2011). Lactic acid also leads to the production of anti-inflammatory cytokine IL-1RA, which inhibits IL-1 receptors and prevents the transmission of pro-inflammatory signals (Aroutcheva et al., 2001), and to the reduction of IL-6 and MIP3-α levels (Arend, 2002; Li et al., 2019). *Lactobacillus* spp. increase TGF-β production, which stimulates the host’s anti-viral response (Smith and Ravel, 2017). They can produce a wide range of bacteriocins, such as gassericin T, acidocin 1F221A, lantibiotic, and bacteriocin IIa, IIc, and J46 (Oscariz and Pisabarro, 2001; Kolls and Linden, 2004; Smith and Ravel, 2017). In the lungs, *Lactobacillus* spp. have also been confirmed to stimulate pulmonary growth and alveolarization process (Singh et al., 2017). Taken together, all these evidences allowed *Lactobacillus* spp. to be properly considered as beneficial microbial species. Protective effects could derive from the anti-inflammatory and immuno-stimulatory properties of lactobacilli (Arpaia et al., 2013; Tooli et al., 2019), whose presence in the respiratory tract may be associated with a reduced incidence of inflammatory airways diseases, such as chronic obstructive pulmonary disease, asthma, hypersensitivity pneumonitis, and eosinophilic or interstitial pneumopathies. Moreover, the development of lungs and of the respiratory tract in general, which can last up to 8 years, could be encouraged by the presence of *Lactobacillus*.

To complete the scenario from a virological point of view, also the presence of TTV was evaluated in both the types of aspirates, because TTV titer has been demonstrated to reflect the individual’s immunological status and to be associated with airways diseases (Bagaglio et al., 2002; Maggi et al., 2003). TTV infection has been associated with an aggravation of inflammatory, kidney, and hepatic diseases (Gallian et al., 1999; Touinssi et al., 2001; Yokoyama et al., 2002; Rocchi et al., 2009). TTV is also considered to have a role in the onset of leukemia and lymphoma (Foschini et al., 2001; de Villiers et al., 2002) and to act as a cofactor in some autoimmune diseases (Sospedra et al., 2005; zur Hausen and de Villiers, 2005). Due to TTV’s numerous transmission routes, more than 50% of the general population is a TTV carrier (Bagaglio et al., 2002). However, most of the infections are believed to occur *in utero* (Arpaia et al., 2013). The absolute quantification of TTV in Real-Time PCR revealed that there were no statistically significant differences between VD and CS samples, thus suggesting that the presence of TTV in the infant’s deep airways is not conditioned by the mode of delivery. This finding supports studies indicating that the contact with TTV presumptively occurs *in utero* or during post-natal life, rather than during delivery.

In conclusion, herein we underline the correlation between mode of delivery and proper colonization and development of a lower airways microbiota in newborns. By passing through the maternal vaginal canal, babies result to be enriched in their neonatal microbial populations, even in remote body districts as the lower respiratory tract. Hence, pregnant women who are asked to decide for vaginal delivery or caesarean section should be properly informed about the actual consequences of these modes of delivery on their children’s health. Medical staff should be also incited to a more appropriate application of CS, limiting it to pathological pregnancies or particular conditions.

## Data Availability Statement

The raw data supporting the conclusions of this article will be made available by the authors, without undue reservation.

## Ethics Statement

The studies involving human participants were reviewed and approved by the Independent Review Boad. Written informed consent to participate in this study was provided by the participants’ legal guardian/next of kin.

## Author Contributions

Conception and design of the study, MP, FM, EG, PM. Methodology, EC, MC, AV, LM, DM, FC, AP. Validation, EG, PM. Formal analysis, EG, PM. Investigation, EC, MC, AV, LM, DM, FC, AP. Writing—original draft preparation, EC, MC. Writing—review and editing, EC, MC, AV, LM, DM, FC, AP, MP, FM, EG, PM. Supervision, EG, PM. All authors contributed to the article and approved the submitted version.

## Conflict of Interest

The authors declare that the research was conducted in the absence of any commercial or financial relationships that could be construed as a potential conflict of interest.

## Publisher’s Note

All claims expressed in this article are solely those of the authors and do not necessarily represent those of their affiliated organizations, or those of the publisher, the editors and the reviewers. Any product that may be evaluated in this article, or claim that may be made by its manufacturer, is not guaranteed or endorsed by the publisher.
